# Multisite Kinetic Modeling of ^13^C Metabolic MR Using [1-^13^C]Pyruvate

**DOI:** 10.1155/2014/871619

**Published:** 2014-12-08

**Authors:** Pedro A. Gómez Damián, Jonathan I. Sperl, Martin A. Janich, Oleksandr Khegai, Florian Wiesinger, Steffen J. Glaser, Axel Haase, Markus Schwaiger, Rolf F. Schulte, Marion I. Menzel

**Affiliations:** ^1^GE Global Research, 85748 Garching bei München, Germany; ^2^Medical Engineering, Tecnológico de Monterrey, 64849 Monterrey, NL, Mexico; ^3^Medical Engineering, Technische Universität München, 85748 Garching bei München, Germany; ^4^Nuclear Medicine, Technische Universität München, 81675 Munich, Germany; ^5^Chemistry, Technische Universität München, 85748 Garching bei München, Germany

## Abstract

Hyperpolarized ^13^C imaging allows real-time *in vivo* measurements of metabolite levels. Quantification of metabolite conversion between [1-^13^C]pyruvate and downstream metabolites [1-^13^C]alanine, [1-^13^C]lactate, and [^13^C]bicarbonate can be achieved through kinetic modeling. Since pyruvate interacts dynamically and simultaneously with its downstream metabolites, the purpose of this work is the determination of parameter values through a multisite, dynamic model involving possible biochemical pathways present in MR spectroscopy. Kinetic modeling parameters were determined by fitting the multisite model to time-domain dynamic metabolite data. The results for different pyruvate doses were compared with those of different two-site models to evaluate the hypothesis that for identical data the uncertainty of a model and the signal-to-noise ratio determine the sensitivity in detecting small physiological differences in the target metabolism. In comparison to the two-site exchange models, the multisite model yielded metabolic conversion rates with smaller bias and smaller standard deviation, as demonstrated in simulations with different signal-to-noise ratio. Pyruvate dose effects observed previously were confirmed and quantified through metabolic conversion rate values. Parameter interdependency allowed an accurate quantification and can therefore be useful for monitoring metabolic activity in different tissues.

## 1. Introduction

While ^13^C magnetic resonance spectroscopy (MRS) has been utilized for* in vivo* imaging and spectroscopy of metabolism [1] for a long time, only the development of dynamic nuclear polarization (DNP) helped to overcome the inherent sensitivity limit; as through hyperpolarization using DNP followed by rapid dissolution, the ^13^C MR signal can be amplified by more than 10,000-fold [2].

One of the most common and viable agents for* in vivo* use is [1-^13^C]pyruvate (PYR) [3]. After intravenous injection, it is transported to the observed tissue or organ under observation, where it is enzymatically metabolized to its downstream metabolites [1-^13^C]alanine (ALA) by alanine transaminase (ALT), [1-^13^C]lactate (LAC) by lactate dehydrogenase (LDH), and [^13^C]bicarbonate (BC) by pyruvate dehydrogenase (PDH) to varying extent, depending on tissue type and predominant metabolic activity. At the same time PYR is in chemical exchange with [1-^13^C]pyruvate-hydrate (PYRH). As part of gluconeogenesis, PYR may also be carboxylated to oxaloacetate [4].

In order to quantify the metabolic exchange between PYR and its downstream metabolites, MRS data acquired over a certain time period after injection first require assignment of spectral peaks [5] in the spectral domain and second require quantification of these peaks over time. Several different methods have been used for this time-domain analysis, and among these the most simple and robust method is the determination of metabolite signal ratios. These ratios are usually obtained from the peak metabolite signals [6] or through integrating over time [5]. The latter approach has been employed in our previous study, conducted by Janich et al. [5], where hyperpolarized PYR spectra were quantified for different PYR doses and subsequently used to determine the dose effects on Wistar rats based on time integrated metabolite signal ratios.

Although the approach of obtaining relative metabolite signal ratios, LAC to PYR or ALA to PYR, is straightforward and robust, independently if obtained from peak signal or time integrals, the results suffer from an increasingly strong *T*_1_ weighting of the integral, which skews the resulting ratios. Furthermore, although time-domain visualization and signal ratio determination is an effective tool for assessing the effect of different PYR doses, it provides no quantitative kinetic data of metabolic exchange.

In order to achieve this quantification, different methods for kinetic modeling of hyperpolarized ^13^C MR data have been reported. Most approaches, derived from the modified Bloch equations, represent a two-site interaction between PYR and one specific downstream metabolite, for example, either LAC or ALA [7–14]. Modeling can be extended to include more sites (intra- and extracellular) or more metabolites [9, 12] (for a comprehensive comparison, see [15]). Even so, presumably for robustness, previous work focuses primarily on fitting data with just one downstream metabolite, keeping most parameters fixed, or even model free, based on signal ratios [5, 16, 17]. When PYR is injected and the corresponding metabolic reactions begin to take place, PYR is not metabolized exclusively into ALA (or LAC), but it changes dynamically into all of the aforementioned downstream metabolites [18]. There is furthermore some skepticism, if the implicit assumption of rate constant stability holds in all applications [17] and there are few analyses on model parameter dependence on SNR [19]. In particular, metabolic conversion in the heart predominantly follows the PDH path producing BC [6, 20]. We therefore hypothesize that the simultaneous consideration of various metabolic pathways is necessary to obtain an accurate evaluation of* in vivo* metabolic conversion rates. On this basis, we propose using a mathematical framework for multisite modeling (similar to [8, 21, 22]) by simultaneously fitting different possible ^13^C metabolic pathways for PYR, which can typically be observed after injection of pyruvate labeled in the [1-^13^C] position.

Additionally, although our prior work [5] evaluates quantification of spectra and employed a semiquantitative method to investigate metabolic conversion under different PYR doses (based on metabolite to PYR ratios), it does not provide fully quantitative kinetic data. Therefore, in this subsequent work we employ the experimental data obtained in [5] and implement the proposed multisite, dynamic model to determine metabolic conversion and signal decay rates for full quantification of the kinetics of metabolic conversion. Furthermore, the proposed model gives access to effective longitudinal relaxation times (*T*_1eff_), both for PYR and for the downstream metabolites.

Using the identical biological data, the kinetic parameters estimated by the multisite model are then compared to the parameters obtained using the two-site models proposed both in [8] and in [23]. The estimated parameters of all models are also compared between the three different doses utilized in [5], that is, 20, 40, and 80 mM (corresponding to 0.1, 0.2, and 0.4 mmol/kg bodyweight) of PYR, in order to evaluate the capability of the model for the assessment of dose response. As identical data is used, the evaluation allows for direct assessment of kinetic model stability and quality. Ideally, a successful kinetic model would allow the reduction of data variability due to modeling to a minimum, allowing the visualization of biological variability (i.e., as a response to dose treatment, etc.). In addition, using simulated metabolic data based on exemplary conversion rates, we assessed the variability and stability of the kinetic models under the influence of noise. Here, the expectation towards a model is that both systematic bias and standard deviation of the resulting metabolic conversion rates should be as low as possible over a large range of signal-to-noise ratio (SNR).

## 2. Theory

In our previous study [5], MRS spectral data after injection of pyruvate was acquired and analyzed utilizing time-domain fitting with AMARES [24], resulting in a time course of metabolite levels. To quantify the metabolic conversion, this previous study employed integrated metabolite signal ratios. In the following paragraphs, we will compare this simple integrative approach to kinetic modeling using three different approaches, which are two-site exchange differential model, two-site exchange integral model, and multisite exchange integral model.

### 2.1. Two-Site Exchange Differential Model

Using a two-site exchange differential model (2SDM) allows computing metabolic exchange rates *k*_pyr→*x*_ and the respective metabolite's effective signal decay rates *r*_*x*_ by solving a system of linear equations given in differential form

(1) $$\begin{array}{c} \frac{dM_{x}\left(t\right)}{dt}=-r_{x}M_{x}\left(t\right)+k_{\text{pyr}\to x}M_{\text{pyr}}\left(t\right). \end{array}$$

The effective metabolite signal decay rate *r*_*x*_ is dominated by *T*_1_ relaxation, the respective backward metabolic exchange rate *k*_*x*→pyr_, and a flip angle (FA) term, which also depends on the repetition time (TR), accounting for the irreversible consumption of signal after successive excitations:

(2) $$\begin{array}{c} r_{x}=\frac{1}{T_{x}}+k_{x\to \text{pyr}}+f\left(\text{FA}\right) \end{array}$$

with

(3) $$\begin{array}{c} f\left(\text{FA}\right)=\frac{1-\cos\left(\text{FA}\right)}{\text{TR}}. \end{array}$$

Hence, *r*_*x*_ results in a single, inseparable term of signal decay. However, FA and TR are known from experimentation and can be corrected for. In case the backward exchange rate *k*_*x*→pyr_ is assumed to be negligible, true *T*_1_ relaxation times can be quantified; however, it remains unclear whether this assumption holds true in all physiological states of the animal.

2SDM does not assume a PYR input function and for that reason the first order differential equation (1) can be solved as a linear system. This approach is independent of the time course of PYR administration and is therefore straightforward to apply.

### 2.2. Two-Site Exchange Integral Model

Another approach in kinetic modeling, the two-site exchange integral model (2SIM), assumes a PYR input function that represents the PYR signal in time (*M*_pyr_(*t*)). In Zierhut et al. [8] a series of piecewise defined exponential equations were presented:

(4) $$\begin{array}{c} M_{\text{pyr}}\left(t\right)=\left\{\begin{array}{cc} \frac{I_{\text{pyr}}}{r_{\text{pyr}}}\left[1-e^{-r_{\text{pyr}}\left(t-t_{\text{arrival}}\right)}\right], & t_{\text{arrival}}\leq t<t_{\text{end}},\\ M_{\text{pyr}}\left(t_{\text{end}}\right)e^{-r_{\text{pyr}}(t-t_{\text{end}})}, & t\geq t_{\text{end}}. \end{array}\right. \end{array}$$

The first part of the equation takes into account PYR signal loss due to *r*_pyr_ and the injection of PYR with a constant rate *I*_pyr_ from the arrival time *t*_arrival_ until *t*_end_. It nevertheless assumes that no conversion of PYR takes place during injection. The second part, for all time measurements later than *t*_end_, is characterized only by the PYR signal loss. In a similar manner, an assumption on the initial PYR concentration can be made instead of an assumption on the input function, leading to the modeling of only the exponential decay, as shown in [25]. Explicit modeling of *M*_pyr_ allows for (1) to be solved yielding metabolite signals in time [8]:

(5) Mxt=kpyr→xIpyrrpyr−rx1−e−rxt−tarrivalrx−1−e−rpyrt−tarrivalrpyr,               tarrival≤t<tend,Mpyrtend∗kpyr→xrpyr−rxe−rxt−tend−e−rpyrt−tend +Mxtende−rxt−tend,               t≥tend.

Alongside the parameters characterizing the PYR input function, these equations contain the same parameters (*k*_pyr→*x*_ and *r*_*x*_) that were solved for using 2SDM.

2SIM can be considered as a two-step approach. First, *t*_arrival_, *r*_pyr_, and *I*_pyr_ are determined by fitting (4) to the measured PYR signal. *t*_end_ is simply calculated by summing *t*_arrival_ and the known injection duration. These parameters are then utilized to fit (5) to the LAC and ALA signals. In [6], this model is also utilized to fit the BC signal. Finally the computed metabolic exchange rates *k*_pyr→*x*_, the decay rate *r*_pyr_, and the flip angle correction (3) can be used to estimate apparent *T*_1_ relaxation of PYR.

### 2.3. Multisite Exchange Integral Model

As described above, the metabolite signal decay rate *r*_*x*_ depends on *T*_1_ relaxation, backward metabolic exchange rates *k*_*x*→pyr_, and signal loss from flip angle variations. On the other hand, the PYR signal decay *r*_pyr_ does not depend on backward metabolic exchange, but on forward metabolic exchange rates *k*_pyr→*x*_. This signifies that the rate of PYR decay is also proportional to the rate of PYR downstream conversion.

Hence, when passing from 2SIM to a multisite exchange integral model (MSIM), the PYR input function (4)—represented in its differential form—needs to include all of the metabolic exchange rates:

(6) $$\begin{array}{c} \frac{{dM}_{\text{pyr}}\left(t\right)}{dt}=\left\{\begin{array}{cc} -r_{\text{pyr}}M_{\text{pyr}}\left(t\right)-\underset{x}{\sum }k_{\text{pyr}\to x}M_{\text{pyr}}\left(t\right)+I_{\text{pyr}}, & \\ \;t_{\text{arrival}}\leq t<t_{\text{end}}, & \\ -r_{\text{pyr}}M_{\text{pyr}}\left(t\right)-\underset{x}{\sum }k_{\text{pyr}\to x}M_{\text{pyr}}\left(t\right), & \\ \;t\geq t_{\text{end}}. & \end{array}\right. \end{array}$$

Note that both the PYR signal decay rate *r*_pyr_ and the sum of all of the metabolic exchange rates ∑_*x*_ *k*_pyr→*x*_ are multiplied by the same term *M*_pyr_(*t*) and can therefore be grouped into a total PYR signal decay rate:

(7) $$\begin{array}{c} R_{\text{pyr}}=r_{\text{pyr}}+\sum_{x}k_{\text{pyr}\to x}. \end{array}$$

By replacing (7) in (6), the integral form of the new PYR input function reads

(8) $$\begin{array}{c} M_{\text{pyr}}\left(t\right)=\left\{\begin{array}{cc} \frac{I_{\text{pyr}}}{R_{\text{pyr}}}\left[1-e^{-R_{\text{pyr}}\left(t-t_{\text{arrival}}\right)}\right], & t_{\text{arrival}}\leq t<t_{\text{end}},\\ M_{\text{pyr}}\left(t_{\text{end}}\right)e^{-R_{\text{pyr}}(t-t_{\text{end}})}, & t\geq t_{\text{end}}. \end{array}\right. \end{array}$$

The representation of the total PYR relaxation rate *R*_pyr_ as the sum of the PYR relaxation rate and the metabolic conversion rates allows for a simultaneous fitting process, where the conversion rates are taken into account also in the PYR input function, creating dependent curves and a parameter interdependency. In addition, the estimation of *T*_1_ values for PYR can be achieved directly using

(9) $$\begin{array}{c} \frac{1}{T_{1\text{pyr}}}=r_{\text{pyr}}-f\left(\text{FA}\right). \end{array}$$

Utilizing the same *R*_pyr_ term for the metabolite signals, (5) becomes

(10) Mxt=kpyr→xIpyrRpyr−rx1−e−rxt−tarrivalrx−1−e−Rpyrt−tarrivalRpyr,               tarrival≤t<tend,Mpyrtend∗kpyr→xRpyr−rxe−rxt−tend−e−Rpyrt−tend +Mxtende−rxt−tend,               t≥tend.

As seen in (2), the backward exchange rates are inseparably confounded with *T*_1_ in the respective signal decay rate *r*_*x*_ of each metabolite. A nonnegligible backward reaction thus leads to an overestimation of the true *T*_1_ values for all of the downstream metabolites. For LAC, the overestimation might be considered negligible since the backward reaction was reported to have only a very small effect on kinetics [26], although earlier work indicates upregulated gluconeogenesis in liver-metabolism of tumor-bearing rats [27]. The assumption of negligible backward reactions might also not hold for ALA. There is no need to apply a backward exchange to BC; however, depending on pH, it is breathed out as ^13^CO_2_ and this could lead to an apparent shortening in *T*_1_. This signifies that the *T*_1_ values for ALA and BC obtained utilizing this model can only be considered bounds for the true value.

## 3. Methods

### 3.1. Experimental Data

The experimental data was obtained from healthy male Wistar rats through the acquisition of slice-selective FID signals in heart, liver, and kidney tissue. Three different hyperpolarized PYR concentrations (20, 40, and 80 mM, which correspond to an injected dose of 0.1, 0.2, and 0.4 mmol/kg bodyweight) were utilized to measure a total of 15 animals. Each dose was injected into five different animals twice, resulting in a total of 10 measurements for each dose. A flip angle of 5° was utilized and TR was triggered to animal breathing yielding an average value of ~1 s. SNR was calculated by dividing the maximum PYR signal by the average noise for all time steps. More experimental details can be directly found in [5].

Further exemplary data to evaluate modeling performance at presence of pathology were obtained from adult female Fischer 344 rats (Charles River, Sulzfeld, Germany) beating subcutaneous mammary adenocarcinomas. The animals' anesthesia was maintained with 1–3% isoflurane in oxygen starting about 1 h before the first injection. During the experiment, the heart rate, temperature, and breathing signal were monitored using an animal monitoring system (SA Instruments, Stony Brook, NY, USA). All ^13^C animal experiments were approved by the regional governmental commission for animal protection (Regierung von Oberbayern, Munich, Germany). Two injections were performed using an 80 mM concentration, allowing for direct comparison. For this set of experiments, a flip angle of 10° was utilized and TR was fixed to 1 s.

### 3.2. Data Processing

The experimental data *y*_*m*,*i*_ with *m* ∈ {lac, ala, pyr, bc} acquired at time steps *t*_*i*_ was fitted to MSIM in a constrained least-squares sense; that is,

(11) $$\begin{array}{c} \underset{\beta }{\min}f\left(\beta \right)\;\;\text{s}.\text{t}.\;\text{lb}\leq \beta \leq \text{ub}, \end{array}$$

with cost function

(12) $$\begin{array}{c} f\left(\beta \right)={\underset{m}{\sum }\underset{i}{\sum }\left(y_{m,i}-M_{m}(t_{i},\beta )\right)}^{2}, \end{array}$$

parameters *β* = [*r*_lac_,…, *r*_bc_, *k*_pyr→lac_,…, *k*_pyr→bc_, *I*_pyr_, *t*_end_], and lower and upper bounds lb and ub, respectively. While *t*_arrival_ was fixed to the time when the PYR signal reached 10% of its maximum peak value, *t*_end_ was set as a fitting parameter accounting for various injection times. On the contrary, the implementation in [8] kept *t*_end_ fixed while fitting for *t*_arrival_. Even though the duration of the injection was known, fixing *t*_arrival_ in function of its peak value and calculating *t*_end_ as a parameter allowed for different delivery and perfusion times. Delivery, perfusion, and export are however not implicitly included in the model. To improve the convergence properties of the optimization, the gradient of the cost function was calculated analytically. The optimization was carried out using the MATLAB function* fmincon* (MathWorks, Natick, MA, USA) employing the* Trust Region Reflective Algorithm* and a function tolerance of 1*E* − 10. The utilized bound constraints were set to physically relevant limits: upper bounds of 0.1 s^−1^ for metabolic conversion rates *k*_pyr→*x*_, since they have been reported to be of a smaller order [8, 23], and of 0.005 s^−1^ for the decay rates *r*_*x*_ (equivalent to a 200 s inverse effective signal decay rate) and lower bounds establishing the positivity of all parameters. Note that the optimization always converged far away from the bounds and they were only implemented for numerical improvement. After optimization, *T*_1_ values were estimated for all metabolites from the effective signal decay rate (see (2) and (9)). Initial conditions were fixed to expected normal parameters; however, randomizing the starting guess in between bounds and performing various iterations yielded comparable results.

Pyruvate-hydrate (PYRH), which is also present in spectroscopy, was not included in the minimization process. The reason for this is that conversion between PYR and PYRH is not enzymatic and we are interested in quantifying metabolic rates that lead to a better understanding of enzymatic activity. Additionally, since chemical exchange with PYRH is instantaneous and almost in equilibrium, including PYR would require adding three extra parameters to the minimization without providing additional information regarding metabolic activity. In fact, if PYRH were to be included, the immediate conversion of PYR to PYRH would lead to an overestimation of the apparent metabolic rate, which in turn would decrease all other parameters intrinsic in *R*_pyr_ leading to an overestimation of *T*_1_ values for PYR.

The same reasoning holds for the exclusion of additional pools. Although the MSIM model can be further extended to include multiple pools [15, 22], including them only adds variables to the minimization with no direct benefit to the determination of enzymatic conversion rates.

## 4. Results

### 4.1. Convergence and Quality of Fit

**Figure 1:**
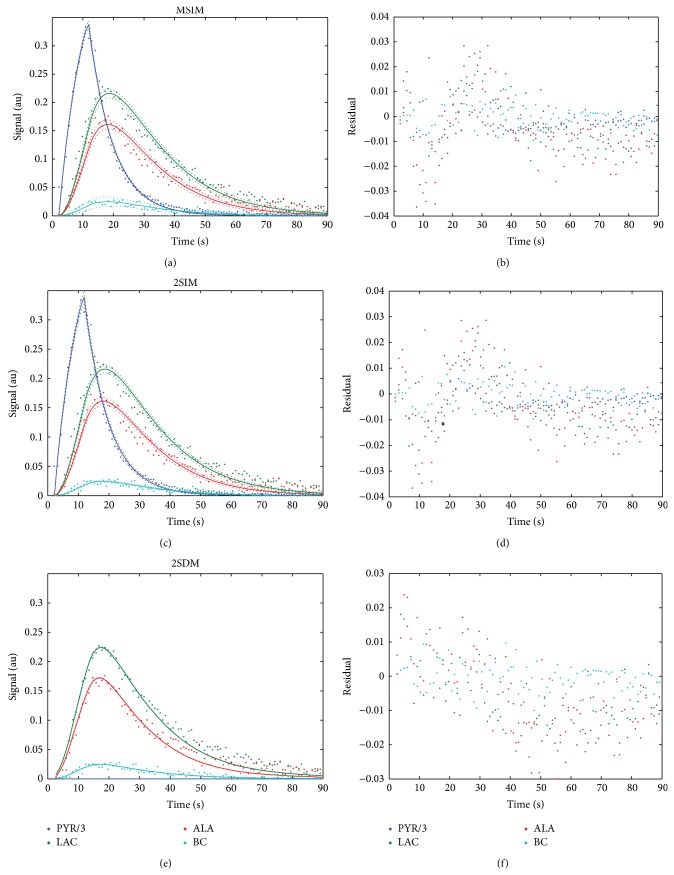
Example of metabolic data acquired for a 40 mM (0.2 mmol/kg) dose in kidney predominant tissue, fitted curves (solid lines) using (a) MSIM, (b) 2SIM, and (c) 2SDM and 95% confidence intervals (dotted lines). (d–f) Residuals of fit.

Parameter fitting with MSIM was shown to converge to an optimal point for every set of experimental data. Figures 1(a)–1(c) show the fitted curves of all metabolites for all models. The residuals for every metabolite and every measurement in the time domain were analyzed (Figures 1(d)–1(f)), and the error of the fitted curves and computed parameters was determined based on the parameter covariance matrix [28]. This error was utilized to determine 95% confidence intervals on the fitted data (see Figures 1(a)–1(c)).

Note that for both MSIM and 2SIM the residuals have a distinct pattern. The pattern indicates that a linear injection rate does not fully model biological activity. In [9], the input function is modeled as a trapezoidal instead of a linear input, but the authors provide no residual analysis. On the other hand, assuming no input function by establishing a fixed initial PYR concentration [25] or solving the differential linear system may not fully account for the entire kinetic time course of the measured signals. In any case, this should be considered as a limitation for both models.

### 4.2. Model Comparison

**Figure 2:**
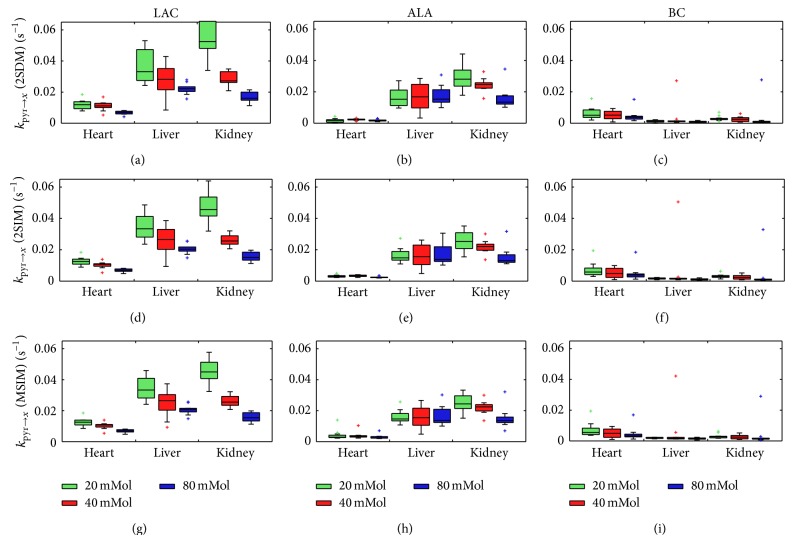
Metabolic conversion rates of LAC (left), ALA (center), and BC (right) obtained for heart, kidney, and liver predominant slices at 20, 40, and 80 mM concentrations (0.1, 0.2, and 0.4 mmol/kg doses) for 2SDM (top), 2SIM (center), and MSIM (bottom). Every box plot displays minima, 25th percentiles, medians, 75th percentiles, maxima, and outliers.

For all of the experimental data, parameters were obtained utilizing the 2SDM, the 2SIM, and the MSIM. While a single implementation of MSIM brought forth parameter values for all downstream metabolites, an independent implementation for LAC, ALA, and BC was necessary in the two-site models. Since all three models were applied on exactly the same experimental data, the comparison between them and to the results obtained for the integrated metabolite signal ratios obtained from Janich et al. [5] directly allows assessing model accuracy separated from biological variability and experiment related inaccuracies like low SNR levels. Results from one exemplary minimization are shown in Table 1; Table 2 displays mean estimated *T*_1pyr_ values for all experiments and their respective SNR levels; and Figure 2 details the obtained metabolic conversion rates for all three models.

Conversion rates and *T*_1PYR_ values calculated with MSIM tended to be lower than those of 2SIM and these in turn are lower than 2SDM (see Tables 1 and 2). Although performance is very similar for all models, reduced data spread can be observed in PYR to LAC conversion in kidney predominant tissue (Figure 2). Since MSIM fits up to nine parameters simultaneously, estimated error from the parameter covariance matrix was usually higher for MSIM.

**Figure 3:**
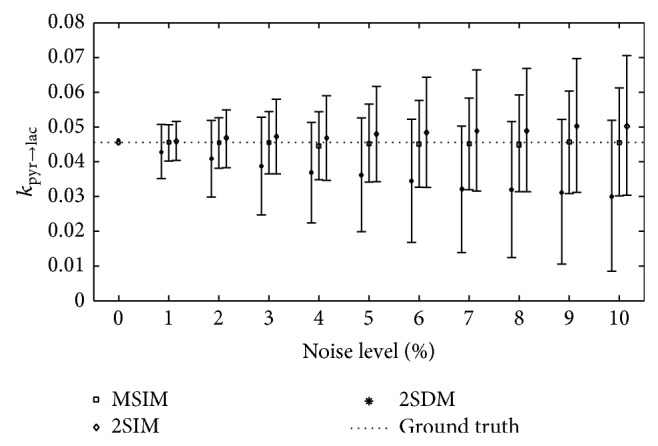
Noise level analysis for exemplary simulated data. Error bars show mean ± standard deviation.

Additionally, for an exemplary dataset, a noise analysis of all three models was implemented by adding Gaussian noise to different extent. Parameters were first obtained from an exemplary minimization with MSIM and were then subsequently used for time curve simulation. Every model was then fit 1,000 times with different initial parameters to this simulated time curve to create a model specific ground truth. Finally, based once again on 1,000 iterations, the simulated dataset was corrupted with random Gaussian noise and minimized with each model.  Figure 3 displays mean and standard deviation of *k*_pyr→lac_ values up to a 10% noise level.

Figure 3 illustrates that although all models yield the same results in noise-free data, with increasing noise both bias and standard deviation of the two-site models 2SIM and 2SDM increase. As a consequence, the resulting metabolic conversion rates obtained from these two-site models increasingly suffer from systematic under- or overestimation. In contrast, the simulation demonstrates that the MSIM model remains bias-free, even with increased noise level, while exhibiting the smallest standard deviation compared to the two-site models.

From experimental results, it is clear that SNR increases with higher concentrations of injected PYR and that 20 mMol injections in liver and kidney predominant tissue had the lowest SNR (with corresponding noise levels of nearly 10%), whereas SNR in heart was generally higher but had a larger standard deviation (Table 2). According to noise simulations, it is precisely in low SNR regions that MSIM is expected to perform with lower deviations. Standard deviations for *T*_1pyr_ values and reduced data spread in 20 mMol *k*_pyr_ quantification, especially in kidney predominant tissue, are indications that this holds.

### 4.3. Pyruvate Dose Assessment

The effects of PYR dose on Wistar rats were examined through the injection of solutions with concentrations of 20, 40, and 80 mM (doses of 0.1, 0.2, and 0.4 mmol/kg) hyperpolarized PYR. Kinetic data was obtained for all downstream metabolites and visualized with the same box plots used in [5]. With this approach, a direct comparison between the results previously obtained and the results obtained with kinetic modeling could be made, using median values as a distance dimension between the results obtained by the different models, rather than as confirmatory values (see Figure 2). As in [5], all median values suggest saturation effects. A more detailed assessment of the PYR dose effects on metabolism and its biological interpretation can be found in [5].

### 4.4. Tumor Evaluation

**Figure 4:**
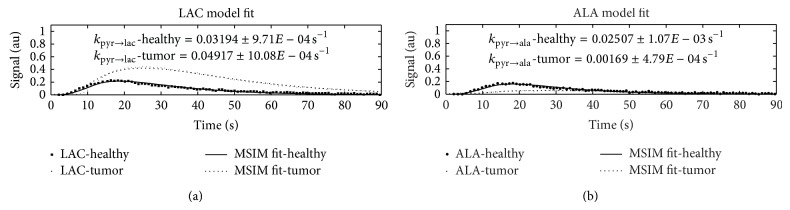
Comparison of *k*_pyr→lac_ and *k*_pyr→ala_ conversion rates between a healthy rat (from an 80 mM dose in kidney predominant tissue) and a rat with mammary carcinoma.

In tumor cells, it is well known that conversion from PYR to LAC is elevated even in the presence of oxygen [29, 30]. Additionally, some tumors show changes in alanine transaminase activity, leading to suppression of conversion of PYR to ALA [31–34]. Both effects were quantified by comparing experimental data obtained from a healthy rat and a rat with mammary carcinoma and using MSIM to obtain conversion rate parameters (see Figure 4). It can be seen that, for the same dose, the *k*_pyr→lac_ conversion rate was more than four times larger in tumor cells than healthy cells and the *k*_pyr→ala_ rate was more than 18 times larger in healthy cells than tumor cells. Therefore, obtained conversion rates provide a quantitative metric of metabolic differences between healthy and tumor cells.

## 5. Discussion and Conclusion

Three different kinetic modeling methods were implemented and investigated for the quantification of time-dependent metabolite levels. The two-site exchange differential model (2SDM) and two-site exchange integral model (2SIM) assume a two-site interaction between pyruvate (PYR) and one specific metabolite. The proposed multisite exchange integral model (MSIM) takes into account various downstream metabolites in one system and allows fitting in a one-step process. That is, all of the parameters are generated in a single minimization, avoiding the need for separate implementations for every specific metabolite and resulting in a robust, optimal convergence far from the imposed constraints.

The three models were compared by taking median values as a distance dimension and, using exemplary simulated data, performing a noise analysis. In this analysis, metabolic exchange rate values obtained with 2SDM and 2SIM showed a bias with increasing noise levels. On the other hand, MSIM showed almost no bias, maintaining the average computed value close to the ground truth even at high noise levels, with smaller standard deviations than 2SDM and 2SIM.

Using the experimental data of  [5], all kinetic models were compared between different PYR concentrations to assess the effect of increased PYR doses on* in vivo* metabolism. Results obtained from all three kinetic models were very similar; however, MSIM yielded smaller data spread for metabolic conversion in low SNR experiments and more accurate effective *T*_1_ values for PYR as downstream metabolite rates are taken into account during the optimization, while effective *T*_1_-estimation in 2SIM requires postprocessing corrections.

MSIM was then further utilized to evaluate model performance in disease. Obtained conversion rates from MSIM showed significant differences in healthy cells in comparison to tumor cells, where LAC conversion was elevated and ALA conversion, on the other hand, was suppressed.

Extending two-site models into a multisite model yields both biological and numerical insight. Biologically, it has been shown that calculated rates give proof of the saturation effects studied in [5] and can be used to quantify metabolic differences between normal and tumor cells. Numerically, a one-step fitting process with parameter interdependency performs marginally better than other fitting methods, particularly in regions with low SNR. Further work with the MSIM model will focus on pixelwise metabolic mapping of cellular activity and its application to different metabolic systems.

## Figures and Tables

**Table 1:** Exemplary parameter estimates (± standard error) obtained from three different kinetic modeling methods for a 40 mM (0.2 mmol/kg) dose of kidney predominant tissue.

Model	MSIM	2SIM	2SDM
*k*_pyr→lac_ [s^−1^]	0.03194 ± 9.71*E* − 04	0.03202 ± 7.75*E* − 04	0.03448 ± 1.15*E* − 03
*k*_pyr→ala_ [s^−1^]	0.02507 ± 1.07*E* − 03	0.02518 ± 4.97*E* − 04	0.02832 ± 1.02*E* − 04
*k*_pyr→bc_ [s^−1^]	0.00379 ± 1.51*E* − 03	0.00381 ± 2.67*E* − 04	0.00392 ± 4.48*E* − 04

*T*_1lac_ [s]	16.36 ± 0.620	16.28 ± 0.488	14.13 ± 0.629
*T*_1ala_ [s]	14.48 ± 0.752	14.38 ± 0.552	12.18 ± 0.578
*T*_1bc_ [s]	14.11 ± 4.78	14.11 ± 1.19	13.46 ± 2.051

*T*_1pyr_ [s]	16.67 ± 0.676	16.82 ± 0.845	N/A^*^

^*^According to (1), 2SDM only fits for *k*_pyr→*x*_ exchange rates and the corresponding *T*_1_ values.

**Table 2:** *T*_1pyr_ calculated for MSIM and 2SIM and corresponding SNR levels for all concentrations and slices (mean ± standard deviation).

	*T*_1PYR_ (MSIM)	*T*_1PYR_ (2SIM)	SNR
20 mMol			
Heart	8.93 ± 2.68	9.04 ± 2.82	15.52 ± 3.87
Liver	22.14 ± 12.26	24.25 ± 14.28	8.62 ± 2.03
Kidney	27.63 ± 12.11	61.61 ± 91.27	11.63 ± 1.87
40 mMol			
Heart	10.02 ± 2.81	10.17 ± 2.88	44.57 ± 15.56
Liver	20.70 ± 3.72	22.83 ± 8.44	20.14 ± 6.36
Kidney	21.11 ± 7.04	21.73 ± 9.20	27.58 ± 5.38
80 mMol			
Heart	10.85 ± 5.98	10.94 ± 6.11	84.65 ± 32.32
Liver	25.75 ± 7.90	25.88 ± 7.89	23.06 ± 14.60
Kidney	20.69 ± 10.38	20.00 ± 10.33	29.61 ± 12.95

## Abbreviations

x: Index of downstream metabolites: lactate, alanine, bicarbonate, and pyruvate-hydrate
$k_{\text{pyr}\to x}$: Metabolic conversion rate from pyruvate to x
LDH: Lactate dehydrogenase
ALT: Alanine transaminase
PDH: Pyruvate dehydrogenase
CA: Carbonic anhydrase
2SDM: Two-site differential model
2SIM: Two-site integral model
MSIM: Multisite integral model
$M_{x}(t)$: Time dependent signal for metabolite x
$M_{\text{pyr}}(t)$: Time dependent pyruvate signal
f(FA): Flip angle function
$t_{\text{arrival}}$: Time of pyruvate arrival
$t_{\text{end}}$: Time at which pyruvate is no longer injected
$I_{\text{pyr}}$: Pyruvate injection rate
$r_{x}$: Metabolite signal decay
$r_{\text{pyr}}$: Pyruvate signal decay rate without metabolic conversion rates
$R_{\text{pyr}}$: Pyruvate signal decay rate including metabolic conversion rates
lb: Vector of lower bounds
ub: Vector of upper bounds
m: Index of all metabolites (lactate, alanine, pyruvate, and bicarbonate)
$t_{i}$: Sampling times
β: Vector of optimization parameters
$y_{m,i}$: Measured data point for metabolite m and time step $t_{i}$
$M_{m}(t,\beta )$: Time dependent signal of metabolite m as a function of parameters β.
